# Perfectionism in Overweight and Obesity: A Scoping Review

**DOI:** 10.1111/obr.70162

**Published:** 2026-05-21

**Authors:** Chantal P. Delaquis, Yasmin Brown, Dean Spirou, Ruby Matthews, Jayanthi Raman, Sylvie Berthoz

**Affiliations:** ^1^ Université de Bordeaux, INCIA CNRS UMR 5287 Bordeaux France; ^2^ Discipline of Psychological Sciences University of Newcastle Callaghan New South Wales Australia; ^3^ Graduate School of Health University of Technology Sydney Sydney New South Wales Australia; ^4^ Department of Psychiatry for Adolescents and Young Adults Institut Médico‐chirurgical Montsouris Paris France

**Keywords:** disordered eating, obesity, overweight, perfectionism

## Abstract

People with a high body mass index (BMI) experience high rates of disordered eating behaviors (DEB) and eating disorders (ED). Perfectionism has been identified as a transdiagnostic mechanism across EDs; however, it is unclear whether it can be considered a transdiagnostic, trans‐BMI mechanism that should be evaluated and targeted in interventions for overweight/obesity. Further, as perfectionism is generally considered to be a multidimensional construct, it is unknown whether certain dimensions may be associated with higher BMI. The present review synthesizes 80 studies that investigated perfectionism in people with overweight/obesity following PRISMA guidelines. Fifty of the included studies investigated clinical samples, and 30 investigated nonclinical samples. The overall mean BMI was 30.38 (SD = 6.13). Although the majority of studies found no direct relationship between perfectionism and BMI, perfectionism appears to be associated with DEB, indicating its potential transdiagnostic relevance. Further, dimensions of perfectionism such as concern over mistakes were linked to eating pathology across weight categories. Our review highlights the need for a consensus on the definition and assessment of multidimensional perfectionism, the lack of qualitative research, and the need to consider the impact of perfectionism on the lived experiences of people with a high BMI.

**Abbreviations**
AN: anorexia nervosa
BMI: body mass index
BN: bulimia nervosa
CBT: cognitive behavior therapy
CBT‐P: cognitive behavior therapy for perfectionism
DBT: dialectical behavior therapy
DEB: disordered eating behaviors
ED: eating disorder
EDI: Eating Disorder Inventory
EDNOS: eating disorder not otherwise specified
FMPS: Frost Multidimensional Perfectionism Scale
GSH: guided self‐help
HC: healthy controls
MDD: major depressive disorder
NSSI: non‐suicidal self‐injury
OB: obesity
ON: orthorexia nervosa
OOP: other‐oriented perfectionism
OW: overweight
RCT: randomized control trial
SOP: self‐oriented perfectionism
SPP: socially prescribed perfectionism
SUD: substance use disorder
TAU: treatment as usual

## 1 Introduction

Overweight and obesity are a major public health crisis due to their growing prevalence, high medical and psychiatric comorbidity, and psychosocial consequences [1, 2, 3]. Current obesity treatments lack efficacy, with the majority of patients regaining weight at follow‐up, even after initial significant weight loss [4]. There is an increasing prevalence of individuals with obesity reporting disordered eating behaviors (DEB) [5]. People with a high body mass index (BMI) are more likely to report DEB and lifetime eating disorders (EDs), except for anorexia nervosa (AN), compared to those who are normal weight [6, 7]. As such, there has been debate over whether EDs and obesity should be considered a spectrum of eating and weight‐related disorders with shared risk factors [8, 9]. Identifying these shared mechanisms may aid in the development of transdiagnostic and trans‐BMI assessments and treatments.

Perfectionism has been identified as a transdiagnostic mechanism across EDs [10]. The leading theoretical models of EDs emphasize the key role of perfectionism, including the transdiagnostic theory of ED [11], the cognitive interpersonal model of AN [12, 13], and the perfectionism model of binge eating [14]. Perfectionism is generally agreed to be a multidimensional construct with two higher‐order factors: perfectionistic concerns and perfectionistic strivings [15]. Perfectionistic concerns reflect a fear of failure/mistakes [16] and perceiving that others demand perfection (socially prescribed perfectionism [SPP] [17]). Perfectionistic strivings can indicate an adaptive focus on achieving goals or the rigid pursuit of excessively high standards [18], as well as demanding perfection from oneself (self‐oriented perfectionism [SOP] [17]).

The majority of perfectionism research has focused on AN and bulimia nervosa (BN); however, given the potential role of perfectionism in binge eating, there has been an increased focus on binge eating disorder (BED) and overweight/obesity [19, 20]. Meta‐analyses have shown that perfectionistic concerns are associated with AN [21, 22] and bulimic symptoms [23]. Evidence for the role of perfectionistic strivings in EDs tends to be less robust than for perfectionistic concerns; however, some evidence suggests that personal standards may be higher in those with restrictive ED profiles than binge/purging profiles [24].

A recent meta‐analysis demonstrated that perfectionism is associated with binge eating, with perfectionistic concerns being linked to more severe binge eating than perfectionistic strivings [20]. It included both clinical and nonclinical samples and focused on binge eating behaviour across EDs, with some studies investigating participants with overweight/obesity. Although binge eating is highly prevalent in overweight/obesity, the mean BMI of the samples was not reported nor considered as a moderator.

Despite decades of research highlighting the importance of perfectionism in EDs, it remains unclear whether perfectionism is a transdiagnostic mechanism that exists across the BMI spectrum and independently of the presence of an ED. Gaining knowledge on this issue is particularly important to guide clinical practice and improve treatments for overweight/obesity.

To our knowledge, no review has summarized the literature on the association between perfectionism and overweight/obesity. Due to the paucity of research on this topic, a scoping review was deemed the most appropriate method to explore a broad range of literature. We aimed to answer the following research questions: (1) What kind of relationship exists between perfectionism and overweight/obesity? (2) Are particular facets of perfectionism related to overweight/obesity?

## 2 Method

This scoping review adhered to the Preferred Reporting Items for Systematic Reviews Extension for Scoping Reviews (PRISMA‐ScR) guidelines [25] and was pre‐registered on Open Science Framework (OSF; available from https://osf.io/mpbcy/).

We searched PubMed, PsycINFO, Web of Science, and Scopus on February 26, 2024, to identify published research that examined perfectionism and overweight/obesity. The search terms were: (“obesity”, “overweight”, “binge eating”, “eating disorder”, “bariatric”) AND perfectionis*. No date restrictions were applied. Binge eating, ED, and bariatric were included to detect studies in clinical populations, as well as studies examining multiple EDs and overweight/obesity. A search validation procedure was conducted, where we identified 10 known studies meeting inclusion criteria and cross‐referenced them with search results. Sixty percent of the studies were captured in the initial search. We conducted an additional Google Scholar search and included the first seven pages of results, as follows: After this, the article titles became irrelevant, and all our known studies were identified.

Eligibility criteria included (1) published in English or French, (2) include a perfectionism outcome measure, and (3) report results concerning perfectionism outcomes for participants with overweight or obesity (BMI > 24.9 kg/m^2^). Studies examining a wide range of BMIs were included as long as specific results were reported for participants with overweight/obesity. Non‐experimental designs, such as theoretical papers, systematic reviews, meta‐analyses, and book chapters, were excluded. As this review aims to explore various types of research, the inclusion criteria on types of evidence sources were deliberately kept broad to include scientific articles and gray literature, with both quantitative and qualitative methodologies.

Due to the large number of identified papers, a team member was added to the project (R.M.) to assist in screening and data extraction. To refine our results in the title and abstract screening stage, we excluded studies that did not mention perfectionism in the abstract, as well as studies investigating athletes, due to the confounding nature of high muscle mass on BMI [26]. The original pre‐registration was updated on October 9, 2024.

Data were extracted by four independent reviewers, with two reviewers for each study. Extracted data included author(s), year of publication, country of publication, sample size, study design, exclusion criteria, gender, psychiatric comorbidities, age, BMI, perfectionism outcome measure used and the mean scores (if reported), and the key findings as pertaining to perfectionism for participants with overweight/obesity. Extracted data were assessed for consensus by the consensus reviewer (C.D.). All screening and extractions were conducted using Covidence Systematic Review Software. Disagreements were discussed until consensus. Missing full texts were sought by contacting the corresponding authors. If the corresponding author could not be contacted, then the article was excluded. The number of records identified and excluded at each step of the literature search is detailed in Figure 1.

**FIGURE 1:**
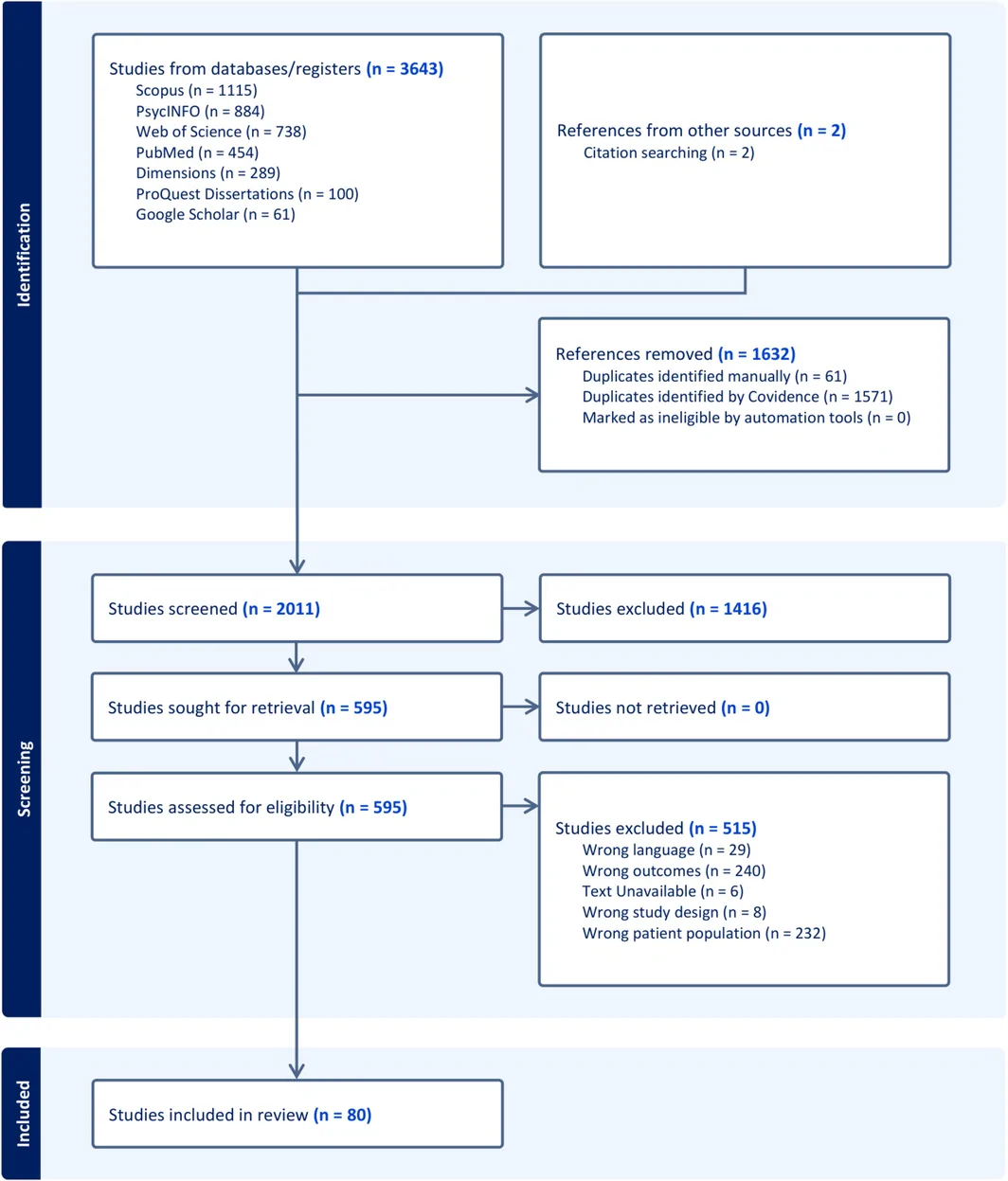
PRISMA flowchart. Flowchart depicting the decision tree of article inclusion and exclusion at each stage of the scoping review.

## 3 Results

### 3.1 Study Characteristics

Eighty studies, published between 1988 and 2024, met inclusion criteria. Seventy were journal articles and 10 were doctoral dissertations. See Table S1 for the full study details. Study methodologies fell into seven broad categories: randomized controlled trial (RCT; *k* = 4), cross‐sectional (*k* = 53), longitudinal (*k* = 9), case–control (*k* = 7), quasi‐experimental (*k* = 6), and mixed methods (*k* = 1). Studies were primarily published in the United States (*k* = 29). The most frequently used perfectionism measures were the Frost Multidimensional Perfectionism Scale (FMPS) (*k* = 30), the EDI (*k* = 14), the EDI‐2 (*k* = 11), and the Hewitt–Flett Multidimensional Perfectionism Scale (HF‐MPS) (*k* = 6).

### 3.2 Sample Characteristics

The sample sizes ranged from 3 to 6007 participants. The majority reported all‐female (*k* = 39) or mostly female (*k* = 33) samples. The mean age was 31.31 years (SD = 10.52; min = 10.85, max = 75.30). The mean BMI across studies was 30.38 (SD = 6.13).^1^ Roughly half the studies included participants with an ED (*k* = 37).

### 3.3 BMI and Perfectionism

Studies were stratified based on sample type (clinical (*k =* 50) or nonclinical (*k* = 30)), then by whether they assessed uni‐ or multidimensional perfectionism. Unidimensional perfectionism was conceptualized as using a total score as the primary perfectionism outcome. Multidimensional perfectionism assessments included subscales (e.g., concern over mistakes and SPP) or maladaptive/adaptive perfectionism. The mean BMI in the clinical samples (*M* = 32.00, SD = 6.56) was significantly higher than the mean BMI in the nonclinical samples (*M =* 27.39, SD = 3.98; *t =* 3.18, *p* < 0.001).

#### 3.3.1 Clinical Samples

##### 3.3.1.1 Unidimensional Perfectionism

Thirty‐three studies included a clinical sample. The majority used the EDI perfectionism subscale total score.

###### 3.3.1.1.1 Group Comparison Studies

Fifteen studies conducted group comparisons where at least one group had overweight and/or obesity. Of these, three directly assessed the correlation between perfectionism and BMI. One found a positive association, although the magnitude of this association was negligible (0.09 [27]), one found a negative association [28], and one found no association [29].

Overall, perfectionism was higher in groups with an ED compared to healthy controls (HC); however, the levels of perfectionism varied depending on diagnosis. Legenbauer and colleagues [30] found that BN (normal weight) and BED (overweight) scored similarly on perfectionism, with both scoring higher than HC. Pignatti and colleagues [31] found that AN and BN (obesity) scored similarly on perfectionism and both scored higher than HC (normal weight). Another study found that BED (obesity) had significantly lower perfectionism than AN but did not differ from HC (normal weight) [32]. Novara and colleagues [33] compared four groups—AN/BN versus obesity/BED versus dieting group (underweight) versus HC (normal weight)—revealing that the AN/BN group scored significantly higher than the other groups on the FMPS total score. Further, they found that perfectionism was associated with orthorexia nervosa (ON) described as an excessive concern regarding healthy food in the obesity/BED and dieting groups. Of note, Jackson and colleagues [34] found that women with BED scored significantly higher on perfectionism than those without BED, but when comparing overweight and normal weight participants, no significant difference in perfectionism was found. When comparing patients with an ED with and without lifetime obesity, there were no significant differences in perfectionism scores, although those with lifetime obesity had greater ED severity and greater overall psychopathology compared to those with an ED and no lifetime obesity [35]. Another study found that perfectionism was positively associated with eating pathology in AN and EDNOS (obesity), but not in BN (normal weight) [36]. In treatment‐seeking patients with overweight/obesity, perfectionism was elevated compared to normal weight patients and controls with overweight [37].

Four longitudinal studies were included. Antecedent perfectionism was associated with the subsequent development of BN symptoms (overweight) [38]. Striegel‐Moore and colleagues [39] found that perfectionism was a general risk factor contributing to vulnerability for BED compared to non‐ED psychiatric controls and HC. Another study demonstrated that males with BN (overweight) had lower perfectionism post‐hospitalization compared to females (normal weight) [40]. One RCT found that dialectical behavior therapy (DBT, compared to control) reduced perfectionism in BED (obesity) and BN (BMI unspecified) [41].

###### 3.3.1.1.2 Exclusively Overweight/Obesity Samples

Fourteen studies included only participants with overweight and/or obesity. Three examined correlations between perfectionism and BMI, and none found an association [42, 43, 44].

Perfectionism was related to symptoms of schizophrenia [45], binge eating severity [46, 47], depressive symptomatology [48], lack of interoceptive awareness [49], overall psychiatric comorbidity, and overall eating pathology [47]. In contrast, one study in treatment‐seeking overweight youth found that higher perfectionism was associated with lower overall ED pathology [50]. Among school‐aged children with overweight/obesity and with or without an ED, perfectionism was positively associated with the presence of an ED [51]. Interestingly, when comparing patients with BED (obesity) to treatment‐seeking individuals with obesity and overeating but no BED, Antoniou and colleagues [52] found no significant differences in perfectionism, with the only significant group difference being the frequency of binge eating. Thurstin et al. [53] categorized participants with obesity into four groups based on the EDI and found that those with high perfectionism presented with binge eating tendencies and rigid approaches to dieting.

Four longitudinal studies were included. Patients at 12‐month post‐sleeve gastrectomy surgery saw improvement on the EDI, body image, and quality of life, but not perfectionism [54]. One found that in treatment‐seeking patients with obesity, changes in uncontrolled eating were more related to changes in depression and perfectionism than to BMI [43]. Further, higher perfectionism and BMI in the first month of a weight‐loss treatment program were associated with greater positive motivation [55]. Another found a marginal reduction in perfectionism (*p* = 0.05) after CBT for patients with overweight/obesity, and changes in perfectionism were associated with changes in the body shape questionnaire and depression, but not weight loss [44].

###### 3.3.1.1.3 Incidentally Overweight/Obesity Samples

Four studies, all longitudinal, investigated a sample that incidentally had participants with overweight/obesity on average. None directly assessed the relationship between BMI and perfectionism. In participants with MDD (overweight), perfectionism decreased following fluoxetine treatment, and changes in perfectionism were positively related to changes in depression severity [56]. A psychoeducation trial for outpatients with comorbid MDD and BN found greater post‐treatment reductions in perfectionism compared to the control group [57]. In patients with an ED (overweight), when comparing enhanced CBT with treatment as usual (TAU), perfectionism decreased in both arms, with no group differences and no influence of the degree of perfectionism on the changes in ED pathology [58]. One study investigating the effects of an Ashtanga yoga intervention for ED using a mixed‐methods approach found that two of three participants reported improvements in perfectionism. Three qualitative themes emerged, including positive embodiment and self‐care, facilitating changes, and helpful features of the yoga classes [59].

##### 3.3.1.2 Multidimensional Perfectionism

Seventeen studies investigated multidimensional perfectionism in a clinical sample. The majority used the FMPS subscales.

###### 3.3.1.2.1 Group Comparison Studies

Nine studies were included. Several dimensions of perfectionism were assessed, including adaptive/maladaptive, clinical perfectionism, concern over mistakes, striving for excellence, and SOP. Only two studies directly assessed the association between perfectionism and BMI. One study found that BMI was positively associated with ED symptomatology but not maladaptive perfectionism, with no significant group differences in perfectionism [60]. However, in another study of women from a private fitness clinic, BMI was positively associated with facets of perfectionism related to personal standards, including striving for excellence, organization, and high standards for others [61]. They also found that women with overweight had significantly higher scores on “high standards for others” compared to women without overweight.

When comparing individuals with BN (normal weight) versus BED (obesity), no differences were found in OOP, SOP, or SPP, although both groups scored higher on SOP than non‐ED participants with obesity [62]. Cella and colleagues [63] found that maladaptive and adaptive perfectionism did not differ between AN, BN (normal weight), and BED (overweight), that maladaptive perfectionism positively correlated with the EDI‐3 ED Risk Composite Score (as did adaptive perfectionism to a lesser extent), and that maladaptive perfectionism (along with body shame and self‐esteem, but not adaptive perfectionism) was associated with eating concerns. Another study comparing ED groups [64] identified two main symptom profiles in BED (obesity): high adaptive and maladaptive perfectionism and low impulsivity (“purely perfectionistic”) or high maladaptive perfectionism and high impulsivity (“emotionally dysregulated”). Of note, most individuals with BED, as well as those with AN‐R, belonged to the “purely perfectionistic” group. Davies and colleagues [65] found among people with BN (overweight), AN, and HC (normal weight) that, overall, concern over mistakes was the FMPS score the most strongly associated with ED pathology. They also found that those with AN had higher concern over mistakes, doubt about actions, and organization than the BN group, with both scoring higher than HC. Although no significant differences between BED (obesity), EDNOS (overweight), and AN were found, clinical perfectionism was associated with dietary restraint and overvaluation of weight and shape [66]. SOP was associated with ED symptoms in AN and EDNOS (obesity), but not with BN (normal weight) [67]. Another study found that women with BED and obesity were more likely to endorse SOP than women with BED without obesity [68].

###### 3.3.1.2.2 Exclusively Overweight/Obesity Samples

Four studies investigated adaptive/maladaptive perfectionism, organization, SOP/OOP/SPP, or categorized participants into perfectionistic subgroups. The direct association between perfectionism and BMI was assessed in a longitudinal study of participants with overweight/obesity who completed CBT, followed by a rhythmic movement therapy for nonresponders to CBT. They found that baseline BMI was positively associated with concern over mistakes/doubt about actions and SOP but not personal standards and OOP and body dissatisfaction was positively associated with both personal standards and concern over mistakes/doubt about actions [69]. Further, rhythmic movement therapy resulted in a decrease in perfectionism and BMI.

Moerk and colleagues [70] found that patients with BED versus depression versus BED/Depression did not differ on OOP, but the depression group scored higher on SOP than HC. Another study found that individuals with overweight/obesity and high (vs. low) SOP and SPP had significantly higher ED pathology, with SPP, as well as positive and negative affect, predicting ED pathology [71].

A longitudinal study examining predictors of dropout from group CBT for weight loss found no differences between completers and non‐completers on any FMPS subscale except for organization, which was higher among the treatment completers [72].

###### 3.3.1.2.3 Incidentally Overweight/Obesity Samples

Four studies were included. One found that greater concern over mistakes was associated with high (vs. low) alexithymia among ED patients [73]. LaMela and colleagues [74] found a link between parental criticism/expectations and bulimic behaviors.

In a longitudinal study investigating telehealth versus in‐person intensive outpatient programs in EDs, perfectionism, ED pathology, and depressive symptoms decreased, and BMI increased in both groups post‐intervention [75]. An RCT of guided self‐help CBT for perfectionism (GSH CBT‐P) versus guided self‐help CBT (GSH CBT) versus placebo for individuals with BN found that concern over mistakes and personal standards decreased in the two intervention groups, as well as in the placebo group [76].

#### 3.3.2 Nonclinical Samples

##### 3.3.2.1 Unidimensional Perfectionism

Nine studies examined unidimensional perfectionism in a nonclinical sample. The majority of studies used the EDI perfectionism subscale total score.

###### 3.3.2.1.1 Group Comparison Studies

Five studies conducted group comparisons where at least one group had overweight and/or obesity. One found that although there was no difference in perfectionism between underweight, normal weight, and overweight participants, perfectionism was higher in those with DEB [77]. Similarly, Edman and colleagues [78] found no group differences between women with and without obesity on perfectionism, although perfectionism correlated with drive for thinness in women without obesity and with self‐loathing in both groups. Graziano and colleagues [79] found no differences in perfectionism between university students with underweight, normal weight, and overweight. In a community sample of women undergoing fertility treatment, women with obesity who were high (vs. low) in perfectionism reported greater shape concern, as well as a higher likelihood of engaging in binge eating; however, clinical perfectionism scores did not differ significantly between weight groups [80]. In contrast, Casagrande and colleagues [81] did find higher perfectionism between students with overweight/obesity compared to normal weight.

###### 3.3.2.1.2 Exclusively Overweight/Obesity Samples

In a study of community participants with overweight/obesity who were taking part in a local weight management program, no difference was found in diet success between participants with high versus low perfectionism, but there was a negative correlation between self‐efficacy and perfectionism [82].

###### 3.3.2.1.3 Incidentally Overweight/Obesity Samples

Three studies were included. Among self‐identified dieters, a path analysis between perfectionism and binge eating found the following: perfectionism → aversive self‐awareness → negative affect → cognitive narrowing → binge eating [83]. Another study investigated female students grouped by ethnicity and found that Black students (overweight) scored higher on perfectionism than White students (normal weight) [84]. In a community sample of participants who engaged in non‐suicidal self‐injury (NSSI) and DEB, those who had engaged in both behaviors scored higher on perfectionism than those who engaged in only one. Perfectionism scores in both groups were well above the norms found in the general population [85].

##### 3.3.2.2 Multidimensional Perfectionism

Twenty‐one studies examined multidimensional perfectionism in a nonclinical sample. The majority used the FMPS subscales.

###### 3.3.2.2.1 Group Comparison Studies

Six studies compared groups where at least one group had overweight and/or obesity. Perfectionism dimensions included adaptive/maladaptive perfectionism, FMPS subscales, SPP, OOP, and SOP, neurotic perfectionism (see [86]), or effortless perfectionism (see [87]). King et al. [88] directly assessed the correlation between BMI and perfectionism, comparing individuals with anorexic tendencies versus bulimic tendencies (overweight) versus weight concerns (overweight) versus no weight concerns (normal weight), finding no association with the FMPS subscales.

Bardone‐Cone et al. [89] compared women who did versus did not feel overweight (i.e., perceived weight status) and found that those who felt overweight with high maladaptive perfectionism reported more bulimic symptoms than those who felt overweight with low maladaptive perfectionism or who did not feel overweight. Of note, perceived weight status emerged as a better predictor of perfectionism than BMI [89]. Iannacconne and colleagues [90] found that among high school students with and without obesity, none of the FMPS subscales predicted ED symptoms. Another study did not find any difference between students with normal weight or overweight/obesity on any FMPS subscale [91]. Pollock‐BarZiv and colleagues [86] found in individuals with Type 1 diabetes that those with overweight/DEB had significantly higher neurotic perfectionism than those with normal weight/no DEB. When comparing students with a high versus low BMI, Ventura et al. [87] found that those who reported high effortless perfectionism engaged in more unhealthy weight control behaviors, but only if they also reported poor body image and had a lower BMI.

###### 3.3.2.2.2 Exclusively Overweight/Obesity Samples

Two studies investigated only participants with overweight/obesity. In a weight control program, adaptive perfectionism did not predict weight loss or program adherence [92]. Among university students with obesity and ON, narcissism and perfectionism were associated with ON symptoms [93].

###### 3.3.2.2.3 Incidentally Overweight/Obesity Samples

Thirteen studies incidentally investigated samples with overweight/obesity on average. Of the seven that directly assessed the association between perfectionism (FMPS concern over mistakes, personal standards and doubt about actions, SOP and SPP, rigid perfectionism, self‐critical perfectionism, and narcissistic perfectionism) and BMI, six found no association [94, 95, 96, 97, 98, 99]. Goel and colleagues [100] found ethnicity‐specific differences in the parental dimensions of perfectionism, where parental criticism and expectations were not related to BMI in White (normal weight), Black (overweight), and Latina (overweight) women but were associated with higher BMI and greater dietary restraint in Asian women (normal weight).

The six remaining studies included SOP, SPP, FMPS subscales, and rigid perfectionism. Among university students, SOP and SPP did not predict DEB when accounting for BMI, age, and affect, although perfectionist cognitions were moderately positively correlated with drive for muscularity among males [101]. In an ecological momentary assessment study in a community sample with DEB, concern over mistakes was positively correlated with dietary restraint, eating concerns, shape, and weight concerns [102]. Among university students, FMPS subscales concern over mistakes, doubt about actions, parental concerns, and expectations predicted body dissatisfaction in males (overweight) but not in females (normal weight) [103]. Luo and colleagues [104] found that, in a community sample with DEB, concern over mistakes, parental criticism, and doubt about actions were moderately positively correlated with binge eating, which was partially mediated by neuroticism. In another community sample, although BMI was not associated with ED symptomatology, the association between maladaptive perfectionism and ED symptoms was partially mediated by aggression [105]. Lastly, rigid perfectionism was positively associated with dietary restriction in a community sample [106].

## 4 Discussion

The present review of 80 studies examined the relationship between perfectionism and BMI, including studies with predominantly cross‐sectional designs, unidimensional measures of perfectionism, and clinical samples.

A total of 17 studies directly assessed the relationship between perfectionism and BMI. Twelve found no association, four found a positive association, and one found a negative association. Of note, the study that found a negative association assessed perfectionism used the control/perfectionism subscale of the Leiden Index of Depression Sensitivity, and it was the only study to use this measure of perfectionism, which may explain these divergent results. Overall, among people living with a higher BMI, perfectionism appears to be associated with ED symptom severity, as well as psychological distress, rather than directly with BMI. In clinical samples, perfectionism was consistently elevated in groups with an ED compared to HC. Notably, the absence of consistent differences between diagnostic groups suggests that perfectionism may be a transdiagnostic factor contributing to disordered eating symptoms across the weight spectrum.

Findings suggest that dimensions of perfectionism, such as concern over mistakes, are linked to ED pathology across weight categories. However, there was high variability in how perfectionism dimensions were measured across studies. There appears to be no clear consensus on how to measure multidimensional perfectionism. For instance, perfectionistic concerns have been conceptualized as concern over mistakes, concern over mistakes/doubt about actions, maladaptive perfectionism, clinical perfectionism (concern over mistakes/personal standards), and SOP. Notably, although there is a general consensus that perfectionism dimensions fall into either perfectionistic concerns or strivings, none of the included studies explicitly mentioned or categorized these dimensions, favoring instead maladaptive versus adaptive perfectionism, or simply referring to the subscale(s). This variability makes comparisons across studies difficult.

The majority of included studies assessed unidimensional perfectionism using the EDI perfectionism subscale, despite perfectionism being widely accepted as a multidimensional construct [107]. A unidimensional conceptualization risks oversimplification, especially considering that this subscale has been shown to have a two‐factor structure that includes SOP and SPP dimensions [108].

Only nine studies assessed the role of perfectionism in treatment, including interventions targeting perfectionism or measuring the associations between changes in primary outcome scores and perfectionism. DBT, psychoeducation, and rhythmic movement therapy were found to reduce perfectionism, although the effect for CBT was marginal and sleeve gastrectomy surgery did not. Additional studies are needed to draw firm conclusions. Meta‐analytic evidence has shown DBT to be an effective treatment for BN/BED [109]. If mechanisms identified in EDs, including perfectionism and emotion dysregulation [110], are also present in overweight/obesity, ED treatments may be adapted and included in weight management interventions.

In studies including only participants with overweight/obesity, there was overall a lack of information on ED status. This is important considering recent debates on distinguishing the condition of clinical obesity from high adiposity [111].

Further, the majority of studies included female participants with North American/European backgrounds, indicating a need for research in sex/gender diverse samples, as well as in different ethnic groups where cultural differences could impact experiences of perfectionism and weight stigma.

## 5 Limitations

Our search strategy was specific to BMI, but there are significant limitations to using BMI as the sole indication of overweight/obesity, and alternative weight‐related metrics should be used alongside BMI [111]. It is also important to note that BMI norms may not be accurate across ethnic groups [112]. Further, although our review did not impose date restrictions to include a broad range of literature, the definition and measure of perfectionism evolved rapidly between 1984 (first version of the EDI), to the development of the FMPS in 1990, to the debates on uni‐ versus multidimensional perfectionism in the early 2000s until today. Therefore, older studies may conceptualize perfectionism differently than studies published in the last decade.

## 6 Implications and Future Directions

Overall, this scoping review suggests that perfectionism appears to be associated with ED symptoms and psychological distress in individuals with a higher BMI and EDs transdiagnostically. Given that most studies found no direct link between perfectionism and BMI, this relationship may be mediated by a third factor, such as emotional eating, binge eating, or body image. Future research should investigate interactions with other putative variables.

Multidimensional assessments should always be used when evaluating perfectionism in both clinical and nonclinical groups. The question remains regarding which type of perfectionism dimensions should be included. Further, a consensus is required on how to group perfectionism subscales. For instance, dimensions could be examined individually before being grouped to form perfectionistic concerns and strivings. We hope that our recommendations to reach a consensus on the definition and assessment of multidimensional perfectionism will allow for future meta‐analytic synthesis.

Studies including participants with overweight/obesity were generally lacking in‐depth information about their clinical status and severity, including ED diagnoses. Efforts should be made to describe the samples' clinical status holistically, as individuals with obesity and an ED/psychological distress may have a different profile than those with high adiposity only.

Our review highlights the lack of qualitative research and the need to expand obesity research on the lived experiences of people with a high BMI. Only one qualitative study was identified. Patient‐centered care is critical to address the stigma towards high BMI that can come from both the clinician and the patient themselves. In order to better understand the role of perfectionism and other potential mediating factors that may contribute to DEB and ED symptoms and their impact on overweight/obesity, we recommend a shift towards integrating patient perspectives.

## Funding

C.D. was supported by the Agence Nationale de la Recherche (Grant Number ANR‐17‐EURE‐0028).

## Conflicts of Interest

The authors declare no conflicts of interest.

## Supporting information


**Table S1:** Summary of included studies.

## Data Availability

The data that support the findings of this study are openly available in Open Science Framework at https://osf.io/mpbcy/files/46t5q.

## Endnotes

^1^
When calculating mean BMI, in the case of multiple groups in one study, only the mean of the overweight/obesity group(s) was retained.
