# Supplementary material for: Perfectionism in Overweight and Obesity: A Scoping Review
Source: Obes Rev. 2026 May 21;27(12):e70162. doi: 10.1111/obr.70162 (PMC13749260; doi:10.1111/obr.70162)
Supplement: Supplementary file 1 [file OBR-27-e70162-s001.pdf]

Supplemental Materials for Article: Perfectionism in Overweight and Obesity: A Scoping Review

Chantal P. Delaquis[1*], Yasmin Brown[2], Dean Spirou[2,3], Ruby Matthews[2], Jayanthi Raman[2], Sylvie Berthoz[1,4]

[1] *Univ. Bordeaux, INCIA CNRS UMR 5287, Bordeaux, France*

[2] *Discipline of Psychological Sciences, University of Newcastle, Callaghan NSW, Australia*

[3] *Graduate School of Health, University of Technology Sydney, Sydney NSW, Australia*

[4] *Dept. of Psychiatry for Adolescents and Young Adults, Institut Mutualiste Montsouris, Paris, France*

Further information can be requested by contacting the corresponding author at: chantal.delaquis@u-bordeaux.fr

The supplementary materials contain the following:

Pages 2-21: Supplemental Table 1. Summary of Included Studies

Pages 22-33: Reference List of Studies Included in the Review

Supplemental Table 1. Summary of Included Studies

Author, year			Sample					Results	
	Country	Design	N	Type	%	Mean Age (SD)	Mean BMI (SD)*	Perfectionism Outcome	Key Findings
**Clinical Studies**									
**Unidimensional Perfectionism**									
Antoniou 2003	Canada	Cross sectional	130	BED vs OB (with overeating)	100%	40.1 (10.1)	41.4 (7.7)	EDI-2	When comparing women with BED to women with OB who overeat, no significant differences in perfectionism were found. The only significant difference between groups on the EDI and Depression/Anxiety measures was the BED group reported more bulimic (binge eating) symptoms than the OB group who overate.
Aragona 2015	Italy	Cross sectional	60	BED with OB and psychosis	91.7%	44.1 (12.2)	38.2 (8.4)	EDI-2	Patients with more severe schizophrenia symptoms reported significantly higher perfectionism.
deJong 2020	The Netherlands	Randomised controlled trial	143	Patients with an ED (AN; BN; BED; OSFED); mean OW	95.8%	27.6 (7.6)	26.0 (6.9)	FMPS (total score)	20 weeks of CBT-E vs TAU for ED; for FMPS, only a significant main effect for time was found but no significant group x time interaction. No differences between conditions were found on reduction of perfectionism. Changes in ED psychopathology were not moderated by the degree of perfectionism.
deZwaan 1994	Austria	Cross sectional	100	BED with OB	100%	39.2 (NA)	35.9 (3.6)	EDI	Perfectionism positively related to binge eating severity.

Dignard 2022	Canada	Dissertation; Quasi-experimental with qualitative	3	Community and ED treatment waitlist; mean OW	100%	45.7 (15.0)	29.5 (NA)	EDI-2	After yoga classes, participants 1 and 3 reported reliable improvements in perfectionism between Times 1 and 3. These changes were clinically significant only for Participant 3. Three qualitative themes: 1. the embodied woman - increased positive embodiment and desire to care for their body 2. processes facilitating changes - exploring changes and processes that pp experienced that facilitated experience of increased positive embodiment 3. helpful features of the classes - identification of elements of the classes facilitating change.
Eddy 2007	United States	Cross sectional	122	OW treatment-seeking youth	55.7%	11.5 (2.3)	25.5 (7.1)	EDI	Increased perfectionism associated with a decrease in overall ED pathology, mediated by thin-ideal internalization and negative affect.
Elizathe 2018	Argentina	Journal; Cross sectional	100	ED vs non-ED; OW/OB school-aged children	37%	10.9 (0.9)	23.3 (4.1)	CPQ$_a$ (total score)	61% OW and 39% OB; Perfectionism significantly different between ED and non-ED groups. Perfectionism associated with presence of an ED.
Fairburn 1998	United Kingdom	Journal; Case-Control	360	ED (BED/BN) vs Psychiatric Control vs HC	100%	24.5 (5.7)	26.9 (5.5)	Childhood Perfection ism	In the BED group, perfectionism was not significant in the regression compared to HC, general psychiatric controls or BN. Childhood traits, particularly negative self-evaluation, perfectionism, shyness, and extreme compliance, all appear to be especially common antecedents of BN. Perfectionism appeared to be especially characteristic of subjects in whom BN subsequently developed.
Fava 1997	United States	Open-label trial	139	*MDD; OW on average*	58.9%	40.6 (10.6)	26.2 (5.0)	EDI	Perfectionism decreased following treatment with fluoxetine. Post-treatment

									changes in depression severity significantly related to changes in perfectionism.
Guisado 2001	Spain	Cross sectional	100	OB undergoing gastric surgery	85%	40.5 (11.2)	NA	EDI	Patients with OB and psychiatric disorders scored higher in Binge Eating, Perfectionism, and global EDI than the non-psychiatric OB group, 18 months post-surgery.
Idini 2023	Spain	Cross sectional	119	ED (OW) vs HC (NW)	93.5%	31.7 (12.0)	25.8 (5.8)	EDI-2	Perfectionism did not correlate with BMI. Stressful life events related to perfectionism only in ED patients compared to HC. Patients with more perfectionism presented more body dissatisfaction and more dissociative experiences.
Iorio 2000	Italy	Cross sectional	141	OW/OB patients, with NW and OW controls	100%	35.1 (9.6)	28.3 (1.4)	EDI	OW outpatients scored significantly higher than OB patients, but similarly to NW patients and OW controls on perfectionism.
Izydorczyk 2013	Poland	Cross sectional	30	BED	100%	24.8 (2.8)	27.2 (5.8)	EDI	Perfectionism negatively correlated with interoceptive awareness but not with actual body image or ideal body image. Increased perfectionism and decreased interoceptive awareness, coupled with low self-evaluation and low interpersonal trust, act as stimuli to binge eating.
Jackson 1996	United States	Cross sectional	128	OW vs NW, with and without BED	100%	48.2 (NA)	NA	EDI-2	Women with BED scored significantly higher on the Perfectionism subscale compared to women who do not binge eat. Perfectionism scores did not differ between the two weight groups.

Klein 2013	United States	Randomised controlled trial	36	Full or subthreshold BED or BN	100%	35.2 (13.7)	NA	EDI	Significant changes in perfectionism in the DBT group compared to control.
Koenen-Woods 2006	United States	Cross sectional	69	OB	78.3%	43.9 (12.1)	48.2 (7.3)	EDI-2	Positive correlation between depressive symptomatology and perfectionism.
Legenbauer 2018	Germany	Longitudinal (perfectionism only at 1 timepoint)	90	BED (OW) vs BN vs HC (NW)	100%	28.1 (7.9)	28.6 (6.8)	YSQ	Both BN and BED had higher scores on unrelenting standards compared to HC but were not significantly different from one another.
Melero 2014	Spain	Non-randomised experimental	46	Morbid OB after sleeve gastrectomy	78.3%	37.0 (NA)	43.0 (5.0)	EDI	Improvement observed in all the analyzed scales, except perfectionism at 12-months post-surgery.
Minkwitz 2019	Germany	Cross sectional	254	OB vs MDD vs HC (NW)	65.4%	38.7 (12.5)	44.2 (5.4)	LIDS – control/perfectionism subscale	Negative correlation of subscales CON with standardised BMI in patients with average BDI-II score. On CON subscale, significant group difference observed between DEP vs HC and DEP vs OB. OBDEP participants had significantly higher CON scores than the OB participants.
Novara 2021	Italy	Cross sectional	329	OB/BED vs AN/BN vs Diet vs HC (UW and NW)	80.2%	37.8 (10.7)	38.9 (5.3)	FMPS (total score)	AN/BN scored statistically significantly higher than other groups in the MPS total score. In the OB/BED group, perfectionistic traits and EDs symptomatology respectively predict ON directly and inversely.
Pignatti 2013	Italy	Case control	60	BN (OB) vs AN vs HC (UW and NW)	100%	28.9 (6.9)	31.9 (4.3)	EDI-2	BN differed from HC, but not from AN on perfectionism. [We can assume BN had higher scores but the descriptive statistics or mean differences are not provided.]
Puttevils 2019	Belgium	Cross sectional	732	BED (OB) vs AN vs BN vs	100%	24.3 (9.1)	32.6 (6.3)	EDI-2	Perfectionism significantly lower in the AN than the BN group. Perfectionism and BMI were significantly and

				EDNOS (UW and NW)					positively correlated, but the magnitude of the association was negligible (0.09)
Qadir 2020	Iraq	Prospective randomized, parallel-group experiment	50	Outpatients with MDD and BN; mean OW	Not given	34.0 (NA)	25.7 (5.2)	EDI	Psychoeducation treatment targeting health education self-esteem and cognitive distortions, resulted in lower perfectionism in the treated group than in the control group, respectively
Rosenberger 2006	United States	Cross sectional	131	Morbid OB	100%	41.8 (10.9)	50.2 (8.2)	EDI	BMI did not correlate with perfectionism. Depression, self-esteem, and perfectionism accounted for 48% of the variance in body image dissatisfaction. Ethnicity, childhood onset of OW, and BMI not significant predictors of body image dissatisfaction.
Rossi 2023	Italy	Cross sectional	421	BED (OB) vs AN vs HC (UW and NW)	82.6%	38.3 (13.4)	41.5 (7.5)	EDI-3	Significant differences between AN, BED and HC. AN had significantly higher perfectionism compared to the general population and BED. No significant difference in perfectionism between BED and HC
Stotland 2005	Canada	Non-randomised experimental	344	OB	100%	41.8 (11.3)	33.7 (6.4)	LOQ	Correlations suggest that changes in uncontrolled eating are more closely related to changes in depression, stress reactions and perfectionism, than to changes in BMI. BMI at time 1 and 2 did not correlate with perfectionism
Stotland 2012	Canada	Longitudinal	6007	OB	90.9%	41.2 (10.8)	33.3 (5.9)	LOQ	Higher perfectionism and log-transformed BMI in the first month of treatment associated with change in positive motivation (thoughts related to the perceived importance or value of weight control). Thus, positive changes (or less negative changes) in weight control were associated with higher

									starting weight, more physical stress and perfectionism.
Striegel-Moore 2005	United States	Journal; Case-Control	321	BED vs Psychiatric Comorbidity Group vs HC	100%	30.6 (6.1)	35.1 (9.7)	ORFI	The combined BED and PC group scored significantly higher than the HC group on perfectionism. Perfectionism appears to be a general risk factor contributing to vulnerability for BED.
Strobel 2019	Germany	Longitudinal	179	BN (mean OW) vs AN	50%	31.0 (11.0)	29.1 (10.1)	EDI	Males with BN had significantly higher BMI than females. At follow-up, 19 (32%) males were OB and 10 females (17%) were OB, with no significant between-group differences. Males had lower perfectionism at follow-up compared to females. Perfectionism slightly but significantly improved from admission to follow-up in males.
Tasca 2011	Canada	Cross sectional	1451	EDNOS (OB) vs AN vs BN (UW and NW)	94.4%	29.0 (9.7)	31.1 (6.7)	EDI-2	Perfectionism had a direct relationship with core ED psychopathology only in EDNOS and AN
Thurstin 1988	United States	Cross sectional	61	OB	90%	38.4 (11.6)		EDI	Four distinct subgroups identified: normal OB type (n = 22), perfectionistic type (n = 18), normal/drive-for-thinness type (n = 12), and distressed'/bulimic type (n = 9). The perfectionistic type presented with tendencies toward binge eating and exacting performance standards. Perfectionistic type patients are dissatisfied with their bodies and want to do something about it with relatively strong perfectionistic tendencies as well as binge-eating propensities, describing rigid approaches to dieting with a subsequent breakdown of impulse control leading to a binge.

									Following such incidents, guilt and dysphoria are experienced with further resolve to "try again." A cyclic pattern of weight loss-weight gain may be seen.
Villarejo, 2012	Spain	Cross-sectional	1383	ED with or without lifetime OB	100%	27.0 (8.3)	NA	EDI	No significant difference between those with an ED and lifetime OB compared to those with an ED and without lifetime OB. Those with lifetime OB had a later age of onset, higher ED severity and greater general psychopathology than those without lifetime OB, a well as a poorer prognosis overall.
Yokoyama 2022	Japan	Longitudinal	165	OW/OB	100%	49.3 (10.5)	23.5 (5.2)	FMPS (total score)	BMI at baseline did not correlate with baseline perfectionism, nor did % weight loss at the end of the intervention. No sig changes in FMPS scores post-intervention.
**Multidimensional Perfectionism**									
Aime 2016	Canada	Cross sectional	134	ED patients; mean OW	100%	35.3 (11.8)	29.9 (8.3)	FMPS	Greater concern over mistakes and lower self esteem among ED patients with high alexithymia vs low alexithymia. Lower BMI in alexithymia group (mean = 27.7) vs non-alexithymia group (mean = 31.0).
Cella 2017	Italy	Cross sectional	80	BED/EDNOS (OW) vs AN vs BN (UW and NW)	100%	25.4 (7.7)	27.5 (5.3)	FMPS	Positive and significant prediction of eating concerns by maladaptive (but not adaptive) perfectionism. Maladaptive perfectionism positively significantly correlated with the EDI-3 ED Risk Composite Score, and so did adaptive perfectionism, to a lesser extent.
Dakanalis 2014	Italy	Cross sectional	403	EDNOS (OW) vs AN vs BN (UW and NW)	98%	25.3 (6.1)	25.9 (4.2)	FMPS	Maladaptive perfectionism mediated insecure attachment and ED symptoms. No group differences across diagnostic groups (AN, BN, EDNOS) in

									perfectionism. BMI was significantly positively linked to ED symptoms but not to maladaptive perfectionism.
Dakanalis 2015	Italy	Cross sectional	679	BED (OB) vs EDNOS (OW) vs AN (NW)	89.5%	28.5 (9.5)	30.3 (7.4)	FMPS	Increased clinical perfectionism was associated with greater dietary restraint. Interpersonal problems, clinical perfectionism and low self-esteem were indirectly associated with dietary restraint through over-evaluation of shape and weight. No structural path differences across the three diagnostic groups.
Davies 2009	UK	Cross sectional	107	BN (OW) vs AN vs HC (UW and NW)	100%	28.0 (8.0)	25.3 (3.8)	FMPS	People with ED report significantly higher levels of perfectionism than HC participants. People with AN have higher levels of perfectionism than the BN group on concern over mistakes, doubts about action and organisation. Overall score on the FMPS is highly correlated with ED pathology with concern over mistakes being the subscale with the highest correlation
Jafari 2016	Iran	Case control	200	With and without OW	100%	31.3 (6.3)	NA	PI	Women who were OW had significantly higher scores on high standards for others subscales as well as the total perfectionism scale compared to women without OW. Striving for Excellence, Organization, Standards for Others, and total perfectionism scale scores had significant positive relationships to BMI
LaMela 2015	Italy	Cross sectional	167	ED patients; Mean OW	95.8%	31.5 (10.4)	25.4 (9.6)	FMPS	Parental criticism and parental expectations dimensions of perfectionism increased the odds of the presence of bulimic behaviours. No

									evidence was found that the causal relationship between weight and shape concerns and bulimic symptoms was mediated by perfectionism dimensions. [Did not investigate the association between perfectionism and BMI, and did not report mean F-MPS scores]
Lampard 2013	Australia	Cross sectional	1451	EDNOS (OB) vs AN vs BN (UW and NW)	94%	29.0 (9.7)	31.3 (10.0)	EDI-2 (split into 2 dimension s)	Self-oriented perfectionism associated with core ED maintaining mechanisms in AN and EDNOS, but not BN.
Levinson 2021	United States	Longitudinal	93	ED patients (AN-R, AN-BP, BN, OSFED, BED, ARFID); mean OW	91.4%	24.9 (8.4)	25.2 (9.1)	FMPS	In-person vs. virtual telehealth exposure-based treatment targeting body image, perfectionism and challenging thoughts with DBT skills; perfectionism scores significantly decreased in both groups. BMI was significantly higher at discharge in both groups. [No analyses on potential associations between BMI and perfectionism].
Malkina-Pykh 2012	Russia	Quasi-Experimental	104	OB	69%	37.6 (6.7)	34.5 (6.4)	PPS; SPS	Conducted a CBT and rhythmic movement intervention for participants with OB. Baseline BMI and body dissatisfaction were positively associated with concern over mistakes/doubts about actions and SOP, but not OOP and personal standards. However, body dissatisfaction correlated positively both with maladaptive and adaptive perfectionism. Rhythmic movement therapy for CBT non-responders resulted in a decrease in all perfectionism outcomes as well as BMI.

Moerk 2002	United States	Case control	113	BED and OB	85.9%	46.8 (12.5)	32.0 (7.7)	HF-MPS; EDI-2	BED, Depressed and BED/Depressed groups did not differ significantly from each other on Other-Oriented perfectionism however, Depressed and HC differed on self-oriented perfectionism. Depressed and BED/Depressed had the highest scores on EDI-2 Perfectionism.
Ostien 2009	United States	Cross sectional	136	BED/OB vs no BED/OB vs AN (UW)	100%	38.8 (NA)	33.0	Modified HF-MPS	Those with BED and high BMI more likely to endorse SOP compared to those with a high BMI without BED.<br>33 high BMI non-BED (51.6%) and 22 high BMI with BED (51.2%) were categorized as somewhat perfectionistic and 14 high BMI non-BED (21.9%), 14 high BMI with BED (32.6%) were categorized as highly perfectionistic.
Peixoto-Placido 2015	Portugal	Cross sectional	276	OW/OB	100%	43.9 (11.9)	32.8 (5.4)	HF-MPS	Participants with high vs. low SOP and SPP had significantly higher ED pathology. SPP predicted eating pathology and eating concerns.
Pratt 2001	United States	Journal; Cross sectional	219	BED (OB) vs. Non-ED (OB) vs. BN (NW)	100%	NA	NA	HF-MPS	The mean scores of the BN and BED groups were not significantly different on any of the subscales. OOP was not associated with BED. SOP was related to general psychiatric symptomatology and none of the ED symptoms present in BED. SPP was related to several ED and general psychiatric measures important to BED, in particular self-esteem. The perfectionism subscale scores for the BED group were not significantly lower than those in the BN group. The non-ED

									group scored significantly lower on SOP than the BN and BED groups did.
Sawamoto 2016	Japan	Randomised controlled trial	119	OB	100%	45.8 (1.7)	31.7 (1.3)	FMPS	Only organization differed between completers vs non-completers. Organization scores were lower for early dropouts (who dropped out during the first half of the weight loss phase, n = 21) compared to late dropouts (who dropped out during the last half of the weight loss phase, n = 8).
Soidla 2020	Estonia	Cross sectional	274	BED (OB) vs AN vs BN vs HC (UW and NW)	100%	24.0 (7.9)	32.5 (4.0)	FMPS	Sorted patients into 5 classes: moderately impulsive, high functioning, purely perfectionistic, emotionally dysregulated, and behaviorally dysregulated.<br>Purely perfectionistic referred to high levels of both maladaptive and adaptive perfectionism and low impulsivity.<br>The Emotionally dysregulated class had the highest maladaptive perfectionism levels, low functional but high dysfunctional impulsivity and high levels of all ED symptoms. A large proportion of BED cases belonged to the purely perfectionistic and emotionally dysregulated class. A large proportion of individuals with BN and BED as well as AN-R belonged to the purely perfectionistic class.
Steele 2008	Australia	Journal; Randomised controlled trial	48	Placebo group mean OW' all meeting diagnostic criteria for BN	97.6%	26.0 (5.8)	25.4 (6.1)	FMPS	At baseline participants in the guided self-help CBT-P focused intervention group reported significantly higher levels of CM compared to those in the guided self-help CBT group (M = 3.23,

									SD = 0.89) and neither of these groups differed significantly from the average reported in the placebo group. Placebo was shown to be as effective as the other two interventions, including for perfectionism scores.
**Non-Clinical Samples**									
**Unidimensional Perfectionism**									
Azzouzi 2019	Morocco	Cross-Sectional	710	Underweight vs NW vs OW vs OB	65.1%	21.3 (2.0)	22.9 (NA)	EDI-2	No significant association between Weight categories and EDI-2 Perfectionism. EDI-2 Perfectionism was higher in subjects with ED-related behavior.
Blackburn 2006	New Zealand	Cross-Sectional	129	Self-identified dieters; mean OW	100%	31.7 (NA)	28.9 (NA)	PANPS	Perfectionism strongly positively predicted aversive self-awareness which, in turn, predicted higher negative affect. Negative affect predicted higher cognitive narrowing, which strongly positively predicted binge eating, although there was also a negative path coefficient between binge eating and cognitive narrowing. [Path analysis for the full sample (mean BMI = 25.54)].
Casagrande 2020	Italy	Case-Control	111	Students with no ED diagnosis; NW vs OW/OB	59.5%	24.5 (3.0)	29.4 (4.3)	EDI-2	There was a significant difference in perfectionism between the NW and the OW/OB.
Edman 2011	United States	Journal; Cross sectional	279	OB vs not OB	100%	25.8 (6.3)	35.7 (3.3)	FMPS (total score)	Perfectionism and anger were similar among college students of both weight groups. There were no group differences on perfectionism. Drive for thinness correlated with perfectionism among the non-OB women. Perfectionism

									correlated with self loathing among OB women and non-OB women.
Edwards 2003	South Africa	Journal; Cross-sectional	80	Female students divided by race; mean OW	100%	20 (NA)	25.6 (4.6)	EDI	Significant difference between black (mean OW) and white (mean NW) participants on perfectionism.
Graziano 2014	United States	Journal; Cross sectional	89	University students; UW vs. NW vs. OW	100%	20.4 (5.8)	NA (36% OW)	FMPS (total score)	In examining the main effect for BMI, no significant differences were found among OW, NW, and UW women on the FMPS. Further, no significant interaction effect was observed.
Kralj 1989	United States	Dissertation; non-experimental pre-post design	40	Local weight management program participants	100%	41.3 (NA)	NA (70% OW)	PS	Significant moderate negative correlation between self-efficacy (but not self-control) and perfectionism total score at time 1. When looking at groups with high and low perfectionism, but no significant differences in diet success were found between groups.
Peyerl 2011	United States	Dissertation; Cross sectional	1219	Community sample recruited from ED and NSSI themed websites; mean OW	93%	27.1 (9.6)	26.8 (8.4)	NPQ (total score)	Participants who engaged in both NSSI and ED behavior scored higher on perfectionism than participants who engaged in only one behavior, either NSSI or ED. There were no group differences between the NSSI and ED group on follow-up analyses. It should be noted that perfectionism means were well above the norms shown for the general population.
Rodino 2016	Australia	Journal; Cross sectional	403	Community sample undergoing fertility treatment; NW vs OW vs OB	100%	34.5 (4.8)	NA (21% OW, 18% OB)	CPQ_b (total score)	Among OB women, perfectionism (driven and critical attitudes) predicted shape concern. Women with OB also had a greater likelihood if engaging in binge eating independent of PCOS status. No significant group differences

									(weight category and presence/absence of PCOS) on CPQ perfectionism scores.
**Multidimensional**									
Bardone-Cone 2009	United States	Journal; Longitudinal	97	African-American women who "feel OW" vs "do not feel OW"	100%	19.0 (1.6)	28.1 (4.7)	HF-MPS; FMPS	Significant interaction effects between maladaptive perfectionism (but not adaptive perfectionism) and perceived weight status at T1 and T2 on bulimic symptoms. Those with high MP and feeling OW had higher bulimic symptoms compared to those who felt OW but had low MP, and those who did not feel OW. Perceived weight status better predictor of perfectionism than BMI.
Barlow 2024	Australia	Journal; Cross sectional	268	Crowdsourced nonclinical community sample; mean OW	53%	36.3 (13.3)	26.0 (6.2)	FMPS	Health anxiety and perfectionism were risk factors for an obsessive focus on healthy eating. Perfectionism and health anxiety positively explained healthy orthorexia and orthorexia nervosa. Perfectionism outcomes did not correlate with BMI.
Byrne 2010	United States	Dissertation; Non-randomised experimental	55	OW in a weight control program	55%	14.8 (1.8)	NA	FMPS	In general, perfectionism was unrelated to weight loss and to adherence to the weight loss program. In this sample, perfectionism appeared to be a poor predictor of weight loss and adherence to the elements of the weight loss program (e.g., self-monitoring).
DiPasquale 2014	United States	Dissertation; Cross sectional	669	Males (OW) and female (NW) music majors	60.2%	20.6 (2.2)	25.3 (5.2)	FMPS	BMI did not correlate with any of the perfectionism dimensions across groups. Concern over mistakes predict disordered eating in all students. Overall, higher BMIs and more concern over

									mistakes were related to bulimic symptoms for males and females.
Downey 2014	United States	Journal; Cross sectional	469	Students; Mean OW (compared males and females)	68.5%	22.4 (6.6)		HF-MPS ; PCI	SOP and SPP did not predict ED pathology when BMI, affect, and age were accounted for. Perfectionistic cognitions moderately correlated with drive for muscularity among males. For females, both SOP and SPP led to behaviors mainly associated with anorexia. In contrast among males, only SOP (and not SPP) led only to behaviors mainly associated with bulimia.
Dryer 2016	Australia	Journal; Cross sectional	158	Community sample; mean OW	100% males	26.9 (5.5)	25.5 (3.8)	HF-MPS	SOP and SPP were not correlated with BMI. Vulnerability to MD and ED depend on pre-existing perfectionistic attitudes, particularly that of SPP. Men who have higher SPP are more likely to interpret sociocultural messages about appearance as standards that need to be met in order to be accepted by family and peers, which motivates them to try to achieve the "perfect body", making them vulnerable to developing both MD and EDs.
Goel 2020	United States	Journal; Cross sectional	1173	Undergraduate students grouped by race/ethnicity mean OW/OB	100%	19.2 (1.7)	26.6 (6.8)	FMPS	PC, and PE were not significantly related to BMI in White, Black (OW), and Latina (OW) women. Greater PE was associated with higher BMI and greater restraint in Asian American (NW) women and greater PC was related to lower BMI and higher body dissatisfaction and frequency of compensatory exercise episodes in

									multiracial women. PC linked to ED symptoms in Black participants (OW).
Han 2022	South Korea and USA	Journal; Cross sectional	692	University students grouped by ethnicity; mean OW	100%	21.0 (3.3)	27.3 (7.0)	DAS	Maladaptive perfectionism did not significantly correlate with BMI. Both attachment anxiety and attachment avoidance were significant predictors for maladaptive perfectionism while controlling the effects of the covariates. BMI covariate was not significant. Insecurely attachment women in the US (OW) were more likely to experience maladaptive perfectionism and diminished self-esteem, which in turn, increased the likelihood of experiencing bulimia.
Hansen 2017	United States	Dissertation; Longitudinal (EMA)	49	Community sample with DEB; mean OW	100%	30.5 (10.1)	28.6 (7.2)	FMPS	Greater negative affect, perfectionism and body dissatisfaction predicted greater eating disordered attitudes and urges. COM perfectionism significantly moderated the association between body dissatisfaction and restraint, eating concerns and behavioral urges. Perfectionism predicted dietary restraint and binge eating.
Iannaccone 2016	Italy	Journal; Cross sectional	222	OB vs non-OB high school students	38.7%	15.5 (1.5)	29.7 (3.4)	FMPS	No perfectionism subscale on the FMPS emerged as a significant predictor of EDI-3 score in a hierarchical regression model that also included parental bonding, self esteem, shame and BMI/gender (separate models for OB and non-OB). Not a significant factor related to ED risk in OB adolescents or non-OB adolescents.

King 2007	United States	Journal; Cross sectional	241	Anorexic tendencies; bulimic tendencies; weight concerns; no weight concerns; 33% current smokers	100%	20.5 (2.4)	26.9 (5.7)	EDI-2; FMPS	BN and weight concerns groups OW; found significant differences in BMI among the four groups. F-MPS total, COM, DA and PS did not correlate with BMI. Mediation analysis showed that PS did not mediate the relationship between bulimic tendencies and smoking status.
Lamanna 2010	United States	Journal; Cross sectional	348	University students; mean OW; Males (OW) vs females (NW)	71%	19.1 (2.0)	26.0 (5.4)	FMPS	For males and females, sociocultural influences about appearance significantly predicted body dissatisfaction, self-esteem, and perfectionism. Perfectionism did not predict body dissatisfaction. COM, DA, PC, and PE each predicted body dissatisfaction in men. Perfectionism did not predict body dissatisfaction in either female sample.
Luo 2013	United States	Journal; Cross sectional	407	Community sample; mean overweight with ED symptoms	47%	38.2 (13.5)	26.4 (6.0)	FMPS	CM, PC and DA were moderately and positively correlated with binge eating, however these associations were partially mediated by neuroticism.
Luszczynska 2015	Poland	Journal; Longitudinal	845	Middle- and high-school students; OW/OB vs NW	59.2%	16.5 (0.9)	NA (24.6% OW/OB)	FMPS	Participants with NW and those with OW/OB did not differ on FMPS subscales.
Martinez 2022	United States	Journal; Cross sectional	336	Community sample; mean OW	47.3%	35.8 (10.0)	25.7 (5.4)	FMPS	Aggression was found to partially mediate the relationship between maladaptive perfectionism and ED pathology for both men and women, but

									there were no differences in the strength of indirect and direct effects, nor for BMI in predicting ED pathology.
Miley 2022	UK	Journal; Cross sectional	670	Community sample; mean OW with ON symptoms	87.8%	39.0 (NA)	28.2 (5.5)	BTPS	Perfectionism and ON did not significantly relate to BMI. Regression revealed both BMI and perfectionism (total score) to predict 7.7% of the variance in ON. Only perfectionism significantly predicted ON, whereas BMI did not. Rigid perfectionism in particular, demonstrated a significant predictive relationship with ON, of a medium effect size.
Oberle 2017	United States	Journal; Cross sectional	459	University students with OB	NA	19.9 (2.8)	39.9 (0.9)	FMPS	For men, higher-BMI individuals compared to lower-BMI individuals, exhibited greater total ON symptomatology. For women, however, the lower-BMI individuals and higher-BMI individuals did not significantly differ on total ON symptomatology. Perfectionism was positively correlated with all ON variables while controlling for gender and BMI. Overall, higher BMI is associated with greater ON symptomatology for men, and narcissism and perfectionism are associated with greater ON symptomatology for both men and women.
Pollock-BarZiv 2005	Canada	Journal; Cross sectional	51	ED (OW) vs. non-ED (NW)	100%	21.5 (3.4)	26.1 (4.6)	NPQ	The ED symptoms group had significantly higher scores for weight preoccupation and neurotic perfectionism, and the mean scores for the subjects without ED symptoms were similar to the values for normal

									perfectionists. Both groups mean values for SPP, OOP, and SOP were similar to those for female university students.
Solomon-Krakus 2020	Canada	Journal; Cross sectional	570	Community sample; mean OW	43.3%	33.9 (9.6)	27.0 (6.3)	PID-5 Rigid Perfectionism	Rigid perfectionism significantly and positively associated with dietary restriction
Thompson 2019	United States	Journal; Cross sectional	97	Community sample of elderly women; mean OW	100%	75.3 (5.2)	25.8 (4.5)	FMPS	Maladaptive perfectionism was not correlated with BMI, but was positively correlated with bulimic symptoms, shape and weight concern, and their impact on daily life. Also negatively correlated with body and face appearance satisfaction. The fact that perfectionism was not correlated with eating pathology could indicate meaningful differences in how older and younger women experience perfectionism.
Ventura 2018	United States	Journal; Longitudinal	376	Students with high vs low BMI	100%	18.5 (0.35)	23.5 (4.3)	CAPS	There was a significant three-way interaction between effortless perfectionism, body image, and BMI, such that the interaction between effortless perfectionism and body image was only significant at low to average BMI, but not at high BMI. Body image moderated the relation between effortless perfectionism and weight control behaviors; specifically, those who endorsed higher levels of effortless perfectionism engaged in more unhealthy weight control behaviors, but only if they also reported poorer body image and with a lower BMI.

*In the case of multiple groups, the mean of the overweight/obesity group(s) is reported.
BMI = Body Mass Index; BTPS = Big Three Perfectionism Scale; CAPS = Child Adolescent Perfectionism Scale; CPQ_a = Child Perfectionism Questionnaire; CPQ_b = Clinical Perfectionism Questionnaire; DAS = Dysfunctional Attitudes Scale; ED = eating disorder; EDI = Eating Disorder Inventory; FMPS = Frost Multidimensional Perfectionism Scale; HC = healthy controls; HF-MPS = Hewitt-Flett Multidimensional Perfectionism Scale; LIDS = Leiden Index of Depression Sensitivity; LOQ = Larocque Obesity Question; NA = Not Available; NPQ = Neurotic Perfectionism Scale; NW = normal weight; OB = obese; ORFI = Oxford Risk Factor Interview; OW = overweight; PANPS = Positive and Negative Perfectionism Scale; PCI = Perfectionistic Cognitions Inventory; PI = Perfectionism Inventory; PID-5 = Personality Inventory for DSM-5; PPS = Personal Perfectionism Scale; PS = Perfectionism Scale; SPS= Social Perfectionism Scale; UW = underweight' YSQ = Young Schema Questionnaire

Reference List of Studies Included in the Review

Aime, A., Cyr, C., Ricard, M.-M., Guevremont, G., & Bournival, C. (2016). Alexithymia and psychopathology in women seeking help for an eating disorder. *Revue Quebecoise de Psychologie*, *37*(1), 115–131.

Antoniou, M., Tasca, G. A., Wood, J., & Bissada, H. (2003). Binge eating disorder versus overeating: A failure to replicate and common factors in severely obese treatment seeking women. *Eating and Weight Disorders - Studies on Anorexia, Bulimia and Obesity*, *8*(2), 145–149. https://doi.org/10.1007/BF03325004

Aragona, M., Petta, A. M., & Balbi, A. (2015). Psychotic phenomena in Binge Eating Disorder: An exploratory MMPI-2 study. *Archives of Psychiatry and Psychotherapy*, *17*(2), 13–20. https://doi.org/10.12740/APP/43321

Azzouzi N, Ahid S, Bragazzi NL, Berhili N, Aarab C, Aalouane R, Boujraf S, & Rammouz I. (2019). Eating disorders among Moroccan medical students: Cognition and behavior. *Psychol Res Behav Manag*, *12*, 129–135. https://doi.org/10.2147/PRBM.S165114

Bardone-Cone, A. M., Weishuhn, A. S., & Boyd, C. A. (2009). Perfectionism and bulimic symptoms in African American college women: Dimensions of perfectionism and their interactions with perceived weight status. *Journal of Counseling Psychology*, *56*(2), 266–275. https://doi.org/10.1037/a0015003

Barlow, I. U., Lee, E., & Saling, L. (2024). Orthorexia nervosa versus healthy orthorexia: Anxiety, perfectionism, and mindfulness as risk and preventative factors of distress. *European Eating Disorders Review*, *32*(1), 130–147. https://doi.org/10.1002/erv.3032

Blackburn, S., Johnston, L., Blampied, N., Popp, D., & Kallen, R. (2006). An Application of Escape Theory to Binge Eating. *European Eating Disorders Review*, *14*(1), 23–31. https://doi.org/10.1002/erv.675

Byrne, S. (2010). *Healthy obsession: The role of personality and self -monitoring in weight loss* (743819657; Issue 3470199) [Fairleigh Dickinson University].

Casagrande, M., Boncompagni, I., Forte, G., Guarino, A., & Favieri, F. (2020). Emotion and overeating behavior: Effects of alexithymia and emotional regulation on overweight and obesity. *Eating and Weight Disorders - Studies on Anorexia, Bulimia and Obesity*, *25*(5), 1333–1345. https://doi.org/10.1007/s40519-019-00767-9

Cella S, Cipriano A, Iannaccone M, & Cotrufo P. (2017). Identifying Predictors Associated with the Severity of Eating Concerns in Females with Eating Disorders. *Res Psychother*, *20*(1), 199. https://doi.org/10.4081/ripppo.2017.199

Dakanalis A, Carrà G, Calogero R, Zanetti MA, Gaudio S, Caccialanza R, Riva G, & Clerici M. (2015). Testing the cognitive-behavioural maintenance models across DSM-5 bulimic-type eating disorder diagnostic groups: A multi-centre study. *Eur Arch Psychiatry Clin Neurosci*, *265*(8), 663–676. https://doi.org/10.1007/s00406-014-0560-2

Dakanalis A, Timko CA, Zanetti MA, Rinaldi L, Prunas A, Carrà G, Riva G, & Clerici M. (2014). Attachment insecurities, maladaptive perfectionism, and eating disorder symptoms: A latent mediated and moderated structural equation modeling analysis across diagnostic groups. *Psychiatry Res*, *215*(1), 176–184. https://doi.org/10.1016/j.psychres.2013.10.039

Davies H, Liao PC, Campbell IC, & Tchanturia K. (2009). Multidimensional self reports as a measure of characteristics in people with eating disorders. *Eat Weight Disord*, *14*(2–3), e84-91. https://doi.org/10.1007/BF03327804

de Jong, M., Spinhoven, P., Korrelboom, K., Deen, M., van der Meer, I., Danner, U. N., van der Schuur, S., Schoorl, M., & Hoek, H. W. (2020). Effectiveness of enhanced cognitive behavior therapy for eating disorders: A randomized controlled trial. *International Journal of Eating Disorders*, *53*(5), 447–457. https://doi.org/10.1002/eat.23239

de Zwaan M, Mitchell JE, Seim HC, Specker SM, Pyle RL, Raymond NC, & Crosby RB. (1994). Eating related and general psychopathology in obese females with binge eating disorder.

Int J Eat Disord, *15*(1), 43–52. https://doi.org/10.1002/1098-108x(199401)15:1<43::aid-eat2260150106>3.0.co;2-6

Dignard, N. A. L. (2022). Embodied and empowered: Evidence for ashtanga yoga as a novel intervention for women with disordered eating. *Dissertation Abstracts International: Section B: The Sciences and Engineering*, *83*(7-B), No-Specified.

DiPasquale, L. D. (2014). Harmony or discord: Disordered eating and personality traits of college music majors. *Dissertation Abstracts International: Section B: The Sciences and Engineering*, *74*(7-B(E)), No-Specified.

Downey CA, Reinking KR, Gibson JM, Cloud JA, & Chang EC. (2014). Perfectionistic cognitions and eating disturbance: Distinct mediational models for males and females. *Eat Behav*, *15*(3), 419–426. https://doi.org/10.1016/j.eatbeh.2014.04.020

Dryer, R., Farr, M., Hiramatsu, I., & Quinton, S. (2016). The role of sociocultural influences on symptoms of muscle dysmorphia and eating disorders in men, and the mediating effects of perfectionism. *Behavioral Medicine*, *42*(3), 174–182. https://doi.org/10.1080/08964289.2015.1122570

Eddy KT, Tanofsky-Kraff M, Thompson-Brenner H, Herzog DB, Brown TA, & Ludwig DS. (2007). Eating disorder pathology among overweight treatment-seeking youth: Clinical correlates and cross-sectional risk modeling. *Behav Res Ther*, *45*(10), 2360–2371. https://doi.org/10.1016/j.brat.2007.03.017

Edman, J. L., Yates, A., Aruguete, M., & Draeger, J. (2011). The impact of self loathing on disordered eating attitudes among obese females. *North American Journal of Psychology*, *13*(3), 539–548.

Edwards, D., d'Agrela, A., Geach, M., & Welman, M. (2003). Disturbances of attitudes and behaviours related to eating in Black and White females at high school and university in South Africa. *Journal of Psychology in Africa; South of the Sahara, the Caribbean, and*

Afro-Latin America, *13*(1), 16–33.

Elizathe LS, Arana FG, & Rutsztein G. (2018). A cross-sectional model of eating disorders in Argentinean overweight and obese children. *Eat Weight Disord*, *23*(1), 125–132. https://doi.org/10.1007/s40519-016-0321-5

Fairburn CG, Doll HA, Welch SL, Hay PJ, Davies BA, & O'Connor ME. (1998). Risk factors for binge eating disorder: A community-based, case-control study. *Arch Gen Psychiatry*, *55*(5), 425–432. https://doi.org/10.1001/archpsyc.55.5.425

Fava M, Abraham M, Clancy-Colecchi K, Pava JA, Matthews J, & Rosenbaum JF. (1997). Eating disorder symptomatology in major depression. *J Nerv Ment Dis*, *185*(3), 140–144. https://doi.org/10.1097/00005053-199703000-00002

Goel NJ, Burnette CB, & Mazzeo SE. (2020). Racial and ethnic differences in the association between parent-oriented perfectionism and disordered eating in college women. *Int J Eat Disord*, *53*(2), 191–200. https://doi.org/10.1002/eat.23179

Graziano, M., & Sikorski, J. F. (2014). Level of disordered eating: Depression, perfectionism, and body dissatisfaction. *Psi Chi Journal of Psychological Research*, *19*(3), 119–128.

Guisado JA, Vaz FJ, López-Ibor JJ Jr, & Rubio MA. (2001). Eating behavior in morbidly obese patients undergoing gastric surgery: Differences between obese people with and without psychiatric disorders. *Obes Surg*, *11*(5), 576–580. https://doi.org/10.1381/09608920160556751

Han, G., Wang, C., Jin, L., & Bismar, D. (2022). Insecure Attachment, Maladaptive Perfectionism, Self-Esteem, Depression, and Bulimic Behaviors for College Women: A Cross-Cultural Comparison. *INTERNATIONAL JOURNAL FOR THE ADVANCEMENT OF COUNSELLING*, *44*(2), 197–219. https://doi.org/10.1007/s10447-021-09462-w

Hansen, M. A. (2017). *Perfectionism as a Moderator of Associations Between Body Dissatisfaction and Disordered Eating: An Ecological Momentary Assessment Study*

(1970364148; Issue 10607942) [Illinois Institute of Technology].

Iannaccone M, D'Olimpio F, Cella S, & Cotrufo P. (2016). Self-esteem, body shame and eating disorder risk in obese and normal weight adolescents: A mediation model. *Eat Behav*, *21*, 80–83. https://doi.org/10.1016/j.eatbeh.2015.12.010

Idini, E., Paredes-Carreño, P., & Valera-Ceamanos, D. (2023). Characteristics of Stressful Life Events and Its Relationship With Body Disperception in Patients With Eating Disorders. *Revista Colombiana de Psiquiatria*. https://doi.org/10.1016/j.rcp.2023.04.003

Iorio, D., Margiotta, N., D'orsi, P., Bellini, O., & Boschi, V. (2000). The Eating Disorder Inventory in evaluation of impaired eating behaviour in subjects requesting nutritional consultation. *Eating and Weight Disorders-Studies on Anorexia, Bulimia and Obesity*, *5*, 206-210.

Izydorczyk, B. (2013). Selected psychological traits and body image characteristics in females suffering from binge eating disorder. *Archives of Psychiatry and Psychotherapy*, *15*(1), 19–33.

Jackson, C. L. (1996). *The effects of binge eating disorder and weight on the Eating Disorder Inventory—2: An exploratory study* (304291010; Issue 9704890) [State University of New York at Buffalo].

Jafari, Z., Khodarahimi, S., & Rasti, A. (2016). Sexual self-esteem and perfectionism in women with and without overweight. *Women & Therapy*, *39*(1–2), 235–253. https://doi.org/10.1080/02703149.2016.1116875

King, L., Saules, K. K., & Irish, J. (2007). Weight concerns and cognitive style: Which carries more "weight" in the prediction of smoking among college women? *Nicotine & Tobacco Research*, *9*(5), 535–543. https://doi.org/10.1080/14622200701188935

Klein, A. S., Skinner, J. B., & Hawley, K. M. (2013). Targeting binge eating through components of dialectical behavior therapy: Preliminary outcomes for individually supported diary

card self-monitoring versus group-based DBT. *Psychotherapy*, *50*(4), 543–552. https://doi.org/10.1037/a0033130

Koenen-Woods, P. J. (2006). *Obesity, depression and eating disorder symptomatology: A complex relationship* (304913209; Issue 3300701) [Argosy University/Hawai'i].

Kralj, D. (1989). Perfectionism, self-efficacy, and self-control as predictors of diet success. *Dissertation Abstracts International*, *50*(4-B), 1649.

La Mela C, Maglietta M, Caini S, Casu GP, Lucarelli S, Mori S, & Ruggiero GM. (2015). Perfectionism, weight and shape concerns, and low self-esteem: Testing a model to predict bulimic symptoms. *Eat Behav*, *19*, 155–158. https://doi.org/10.1016/j.eatbeh.2015.09.002

Lamanna, J., Grieve, F. G., Pitt Derryberry, W., Hakman, M., & McClure, A. (2010). Antecedents of eating disorders and muscle dysmorphia in a non-clinical sample. *Eating and Weight Disorders*, *15*(1–2), e23–e33. https://doi.org/10.1007/BF03325277

Lampard AM, Tasca GA, Balfour L, & Bissada H. (2013). An evaluation of the transdiagnostic cognitive-behavioural model of eating disorders. *Eur Eat Disord Rev*, *21*(2), 99–107. https://doi.org/10.1002/erv.2214

Legenbauer T, Radix AK, Augustat N, & Schütt-Strömel S. (2018). Power of Cognition: How Dysfunctional Cognitions and Schemas Influence Eating Behavior in Daily Life Among Individuals With Eating Disorders. *Front Psychol*, *9*, 2138. https://doi.org/10.3389/fpsyg.2018.02138

Levinson CA, Spoor SP, Keshishian AC, & Pruitt A. (2021). Pilot outcomes from a multidisciplinary telehealth versus in-person intensive outpatient program for eating disorders during versus before the Covid-19 pandemic. *Int J Eat Disord*, *54*(9), 1672–1679. https://doi.org/10.1002/eat.23579

Luo J, Forbush KT, Williamson JA, Markon KE, & Pollack LO. (2013). How specific are the

relationships between eating disorder behaviors and perfectionism? *Eat Behav*, *14*(3), 291–294. https://doi.org/10.1016/j.eatbeh.2013.04.003

Luszczynska, A., Zarychta, K., Horodyska, K., Liszewska, N., Gancarczyk, A., & Czekierda, K. (2015). Functional perfectionism and healthy behaviors: The longitudinal relationships between the dimensions of perfectionism, nutrition behavior, and physical activity moderated by gender. *Current Issues in Personality Psychology*, *3*(2), 84–93. https://doi.org/10.5114/cipp.2015.52085

Malkina-Pykh, I. G. (2012). Effectiveness of rhythmic movement therapy for disordered eating behaviors and obesity. *The Spanish Journal of Psychology*, *15*(3), 1371–1387. https://doi.org/10.5209/rev_SJOP.2012.v15.n3.39422

Martinez K, Guan C, & Walker DC. (2022). I'm angry that I'm not perfect! Aggression mediates the relationship between perfectionism and eating pathology in men and women. *Eat Weight Disord*, *27*(5), 1895–1901. https://doi.org/10.1007/s40519-021-01319-w

Melero Y, Ferrer JV, Sanahuja A, Amador L, & Hernando D. (2014). Psychological changes in morbidly obese patients after sleeve gastrectomy. *Cir Esp*, *92*(6), 404–409. https://doi.org/10.1016/j.ciresp.2013.11.003

Miley, M., Egan, H., Wallis, D., & Mantzios, M. (2022). Orthorexia nervosa, mindful eating, and perfectionism: An exploratory investigation. *Eating and Weight Disorders*, *27*(7), 2869–2878. https://doi.org/10.1007/s40519-022-01440-4

Minkwitz, J., Scheipl, F., Cartwright, L., Campbell, I. C., Chittka, T., Thormann, J., Hegerl, U., Sander, C., & Himmerich, H. (2019). Why some obese people become depressed whilst others do not: Exploring links between cognitive reactivity, depression and obesity. *Psychology, Health & Medicine*, *24*(3), 362–373. https://doi.org/10.1080/13548506.2018.1524153

Moerk, K. C. (2002). *Personality in binge eating disorder and depression: Do similarities in*

personality traits partially account for comorbidity findings? (305495018; Issue 3067577) [State University of New York at Stony Brook].

Novara C, Maggio E, Piasentin S, Pardini S, & Mattioli S. (2021). Orthorexia Nervosa: Differences between clinical and non-clinical samples. *BMC Psychiatry*, *21*(1), 341. https://doi.org/10.1186/s12888-021-03348-2

Oberle, C. D., Samaghabadi, R. O., & Hughes, E. M. (2017). Orthorexia nervosa: Assessment and correlates with gender, BMI, and personality. *Appetite*, *108*, 303–310. https://doi.org/10.1016/j.appet.2016.10.021

Ostien, M. C. (2009). *Similarities and differences in females with regards to perfectionism in those with anorexia nervosa, high BMI (binge eaters vs. Non-binge eaters), and those seeking a healthier lifestyle* (304996765; Issue 1469340) [Southern Illinois University at Carbondale].

Peixoto-Plácido C, Soares MJ, Pereira AT, & Macedo A. (2015). Perfectionism and disordered eating in overweight woman. *Eat Behav*, *18*, 76–80. https://doi.org/10.1016/j.eatbeh.2015.03.009

Peyerl, N. L. (2011). A comparison of the functions of eating disorder behaviors to non-suicidal self-injury. *Dissertation Abstracts International: Section B: The Sciences and Engineering*, *72*(4-B), 2444.

Pignatti, R., & Bernasconi, V. (2013). Personality, clinical features, and test instructions can affect executive functions in eating disorders. *Eating Behaviors*, *14*(2), 233–236. https://doi.org/10.1016/j.eatbeh.2012.12.003

Pollock-BarZiv SM & Davis C. (2005). Personality factors and disordered eating in young women with type 1 diabetes mellitus. *Psychosomatics*, *46*(1), 11–18. https://doi.org/10.1176/appi.psy.46.1.11

Pratt EM, Telch CF, Labouvie EW, Wilson GT, & Agras WS. (2001). Perfectionism in women

with binge eating disorder. *Int J Eat Disord*, *29*(2), 177–186. https://doi.org/10.1002/1098-108x(200103)29:2<177::aid-eat1007>3.0.co;2-x

Puttevils, L., Vanderhasselt, M. A., & Vervaet, M. (2019). Investigating transdiagnostic factors in eating disorders: Does self-esteem moderate the relationship between perfectionism and eating disorder symptoms? *European Eating Disorders Review : The Journal of the Eating Disorders Association*, *27*(4), 381–390. https://doi.org/10.1002/ERV.2666

Qadir, S., & Muhsin, S. (2020). Administration of Psychoeducational Treatment Programs Improves Depression, Eating Disorders, and Associated Psychological Traits. *International Journal of Medical Research and Health Sciences*, *9*(2), 32–42.

Rodino IS, Byrne S, & Sanders KA. (2016). Obesity and psychological wellbeing in patients undergoing fertility treatment. *Reprod Biomed Online*, *32*(1), 104–112. https://doi.org/10.1016/j.rbmo.2015.10.002

Rosenberger PH, Henderson KE, & Grilo CM. (2006). Correlates of body image dissatisfaction in extremely obese female bariatric surgery candidates. *Obes Surg*, *16*(10), 1331–1336. https://doi.org/10.1381/096089206778663788

Rossi, A., Pietrabissa, G., Tagliagambe, A., Scuderi, A., Montecchiani, L., Castelnuovo, G., Mannarini, S., & Dalla Ragione, L. (2023). Many Facets of Eating Disorders: Profiling Key Psychological Features of Anorexia Nervosa and Binge Eating Disorder. *Behavioural Sciences*, *13*(3). https://doi.org/10.3390/bs13030276

Sawamoto, R., Nozaki, T., Furukawa, T., Tanahashi, T., Morita, C., Hata, T., Komaki, G., & Sudo, N. (2016). Predictors of dropout by female obese patients treated with a group cognitive behavioral therapy to promote weight loss. *Obesity Facts*, *9*(1), 29–38. https://doi.org/10.1159/000442761

Soidla, K., & Akkermann, K. (2020). Perfectionism and impulsivity based risk profiles in eating disorders. *International Journal of Eating Disorders*, *53*(7), 1108–1119.

https://doi.org/10.1002/eat.23285

Solomon-Krakus S, Uliaszek AA, & Bagby RM. (2020). Evaluating the associations between personality psychopathology and heterogeneous eating disorder behaviors: A dimensional approach. *Personal Disord*, *11*(4), 249–259. https://doi.org/10.1037/per0000358

Steele AL & Wade TD. (2008). A randomised trial investigating guided self-help to reduce perfectionism and its impact on bulimia nervosa: A pilot study. *Behav Res Ther*, *46*(12), 1316–1323. https://doi.org/10.1016/j.brat.2008.09.006

Stotland, S. C., & Larocque, M. (2005). Early treatment response as a predictor of ongoing weight loss in obesity treatment. *British Journal of Health Psychology*, *10*(4), 601–614. https://doi.org/10.1348/135910705X43750

Stotland, S., Larocque, M., & Sadikaj, G. (2012). Positive and negative dimensions of weight control motivation. *Eating Behaviors*, *13*(1), 20–26. https://doi.org/10.1016/j.eatbeh.2011.10.003

Striegel-Moore RH, Fairburn CG, Wilfley DE, Pike KM, Dohm FA, & Kraemer HC. (2005). Toward an understanding of risk factors for binge-eating disorder in black and white women: A community-based case-control study. *Psychol Med*, *35*(6), 907–917. https://doi.org/10.1017/s0033291704003435

Strobel C, Quadflieg N, Naab S, Voderholzer U, & Fichter MM. (2019). Long-term outcomes in treated males with anorexia nervosa and bulimia nervosa-A prospective, gender-matched study. *Int J Eat Disord*, *52*(12), 1353–1364. https://doi.org/10.1002/eat.23151

Tasca GA, Presniak MD, Demidenko N, Balfour L, Krysanski V, Trinneer A, & Bissada H. (2011). Testing a maintenance model for eating disorders in a sample seeking treatment at a tertiary care center: A structural equation modeling approach. *Compr Psychiatry*, *52*(6), 678–687. https://doi.org/10.1016/j.comppsych.2010.12.010

Thompson KA & Bardone-Cone AM. (2019). Disordered eating behaviors and attitudes and their

correlates among a community sample of older women. *Eat Behav*, *34*, 101301. https://doi.org/10.1016/j.eatbeh.2019.05.004

Thurstin AH, Weinsier RL, Linton PH, & Crist DA. (1988). An Eating Disorder Inventory-based typology of outpatient obese individuals. *J Subst Abuse*, *1*(1), 45–53. https://doi.org/10.1016/s0899-3289(88)80007-5

Ventura, L. M., Randall, E. T., Shapiro, J. B., Kirsch, A. C., Conley, C. S., & Bohnert, A. M. (2018). Looking good and making it seem easy: A prospective study of effortless perfectionism, body image, and BMI in unhealthy weight control behaviors among female adolescents and young adults. *Emerging Adulthood*, *6*(5), 327–335.

Villarejo, C., Fernández-Aranda, F., Jiménez-Murcia, S., Peñas-Lledó, E., Granero, R., Penelo, E., Tinahones, F. J., Sancho, C., Vilarrasa, N., Montserrat-Gil de Bernabé, M., Casanueva, F. F., Fernández-Real, J. M., Frühbeck, G., De la Torre, R., Treasure, J., Botella, C., & Menchón, J. M. (2012). Lifetime Obesity in Patients with Eating Disorders: Increasing Prevalence, Clinical and Personality Correlates. *European Eating Disorders Review*, *20*(3), 250–254. https://doi.org/10.1002/erv.2166

Yokoyama H, Nozaki T, Nishihara T, Sawamoto R, Komaki G, & Sudo N. (2022). Factors associated with the improvement of body image dissatisfaction of female patients with overweight and obesity during cognitive behavioral therapy. *Front Psychiatry*, *13*, 1025946. https://doi.org/10.3389/fpsyt.2022.1025946
